# The Expression of the Senescence-Associated Biomarker Lamin B1 in Human Breast Cancer

**DOI:** 10.3390/diagnostics12030609

**Published:** 2022-02-28

**Authors:** Tareq Saleh, Ahmad Alhesa, Mohammed El-Sadoni, Nisreen Abu Shahin, Elham Alsharaiah, Sofian Al Shboul, Heyam Awad, Sarah Bloukh, Mahmoud Al-Balas, Mohammad Alsalem, Bilal Azab, Tariq N. Aladily

**Affiliations:** 1 Department of Basic Medical Sciences, Faculty of Medicine, The Hashemite University, Zarqa 13133, Jordan; sofian@hu.edu.jo; 2 Department of Pathology, Microbiology and Forensic Medicine, School of Medicine, The University of Jordan, Amman 11942, Jordan; ahm8171602@ju.edu.jo (A.A.); mhm8191130@ju.edu.jo (M.E.-S.); n.abushahin@ju.edu.jo (N.A.S.); h_awad@ju.edu.jo (H.A.); sar0153498@ju.edu.jo (S.B.); ba2659@cumc.columbia.edu (B.A.); tnaladily@ju.edu.jo (T.N.A.); 3 Department of Pathology, King Hussein Medical Center, Royal Medical Service, Amman 11942, Jordan; elham.mashhor@gmail.com; 4 Department of General and Specialized Surgery, Faculty of Medicine, The Hashemite University, Zarqa 13133, Jordan; mahmoud_albalas@hu.edu.jo; 5 Department of Anatomy and Histology, School of Medicine, The University of Jordan, Amman 11942, Jordan; m_alsalem@ju.edu.jo; 6 Department of Pathology and Cell Biology, Columbia University Medical Center, New York, NY 10032, USA

**Keywords:** lamin B1, immunohistochemistry, breast cancer, senescence

## Abstract

Senescence is a major response to cancer chemotherapy and has been linked to unfavorable therapy outcomes. Lamin B1 is a component of the nuclear lamina that plays a pivotal role in chromatin stability. Downregulation of lamin B1 represents an established biomarker for cellular senescence. However, the protein expression level of lamin B1 in malignant tissue, particularly of the breast, has not been previously described. In this work, we investigated lamin B1 protein expression in normal breast epithelium, malignant breast tissue (including adjacent non-malignant tissue) and in malignant tissue exposed to neoadjuvant chemotherapy (NAC) using immunohistochemistry (IHC) in three patient groups (*n* = 15, *n* = 87, and *n* = 43, respectively). Our results indicate that lamin B1 mean positive expression was 93% in normal breast epithelium and 88% in malignant breast cells, but significantly decreased (mean: 55%, *p* < 0.001) in malignant breast tissue after exposure to NAC, suggestive of senescence induction. No significant association between lamin B1 expression and other clinicopathological characteristics or survival of breast cancer patients was recorded. To our knowledge, this is the first report that established the baseline protein expression level of lamin B1 in normal and malignant breast tissue, and its reduction following exposure to chemotherapy. In conclusion, lamin B1 downregulation can be used reliably as a component of multiple biomarker batteries to identify therapy-induced senescence (TIS) in clinical cancer.

## 1. Introduction

Breast cancer is one of the most prevalent types of human malignancies, comprising 30% of the estimated newly diagnosed female cancers in 2021 and the second leading cause of cancer-related deaths in women worldwide [1]. Fortunately, the overall survival rates for breast cancer have improved significantly due to the latest advances in early screening, molecular and pathologic diagnosis as well as the development of effective therapeutic modalities. Nevertheless, further efforts are needed to decrease the morbidity and mortality associated with breast cancer. In particular, the identification of novel drug targets and biomarkers that predict the outcome of currently available therapy is sought.

Cellular senescence has been increasingly recognized as a central component of tumor biology and a fundamental response to anticancer therapies in preclinical studies, hence the name therapy-induced senescence (TIS) [2,3]. Senescence represents a state of stable growth arrest that prevents further replication of tumor cells. Recent evidence strongly argues that TIS might not be a favorable outcome of cancer therapy [4]; however, this largely remains undetermined based on the current lack of understanding of in vivo (clinical) senescence in patients receiving cancer therapy [5]. In addition, the recently evolving literature on the deleterious effects of senescent cells accumulation in response to cancer therapy has generated the premise that senolytics, drugs that selectively eliminate cancer cells, could have utility as adjuvants to conventional cancer therapy [6]. Accordingly, the identification of TIS in breast cancer patients following exposure to therapy is key for an individualized therapy approach.

The senescent phenotype is complex and encompasses a variety of hallmarks including structural changes [7], metabolic dysregulation [8], increased lysosomal biogenesis (marked by the upregulation of the senescence-associated beta-galactosidase, SA-β-gal) [9,10], epigenetic signatures [11], and wide alterations in gene expression resulting in the production and secretion of various secreted proteins, collectively termed as the senescence associated secretory phenotype (SASP) [12]. As part of the structural changes, senescent cells undergo reorganization of their nuclear profile [13]. Lamin B1 is a nuclear intermediate filament protein located within the inner surface of the nuclear envelope and is encoded by the *LMNB1* gene located on chromosome 5q23 in humans [14]. Lamin B1 plays a key role in nuclear stability, DNA replication, gene transcription, cell proliferation, aging, and in the response to oxidative stress [15,16,17,18]. More importantly, increased lamin B1 expression enhances cell migratory potential, and thus it might contribute to cancer progression and metastasis [19,20]. Interestingly, the degradation of lamin B1 is considered a component of nuclear envelope remodeling that occurs as a cell undergoes senescence and is now utilized as an established biomarker for the identification of senescent cells in vitro [21,22]. Subsequently, the downregulation of lamin B1 can potentially be used to identify the induction of TIS in breast cancer patients following exposure to treatment.

While several studies had established the expression of lamin B1 in several malignant tumors including prostate, renal, pancreatic, lung, gastric and hepatocellular carcinomas [23,24,25,26,27], the protein expression level of lamin B1 in breast cancer tissue relative to its expression in the normal mammary epithelium is not established. In that, lamin B1 expression was only explored at the message level, as a previous study showed that lower lamin B1 mRNA expression in breast cancer is associated with worse clinical outcomes [23]. Similarly, Garvalov et al. observed that the complete absence of lamin B1 expression in malignant lung tissue is associated with a worse prognosis, rapid tumor progression and reduced survival rate [28]. In contrast, a recent study revealed that lamin B1 overexpression is significantly associated with poor clinical outcomes in clear-cell renal cell carcinoma [27]. This variability in lamin B1 expression and its association with clinical outcomes suggest that it has different contributions to cancer progression that appear to be cancer type specific [29]. Accordingly, there is a need to evaluate lamin B1 expression in different malignancies, especially since it has been proposed as drug target for certain anticancer therapies (for example, the use of botulinic acid for pancreatic cancer treatment) [25].

Overall, our study is designed to investigate the expression rate of lamin B1 in normal breast epithelium, primary breast invasive carcinoma tissue that was not exposed to senescence-inducing therapy and in primary breast invasive carcinoma tissue that was exposed to senescence-inducing chemotherapy. Data from this report is of relevance to (*i*) the routine identification of lamin B1 in breast cancer tissue using immunohistochemistry (IHC), (*ii*) the study of TIS induction in breast cancer tissue as a consequence of exposure to cancer chemotherapy, and (*iii*) determining the contribution of lamin B1 to clinical outcomes of breast cancer treatment.

## 2. Results

### 2.1. Patients Clinicopathological Characteristics

The total number of patients in this study is 145 (*n* = 145). Group A represents patients with normal breast epithelium (*n* = 15, where bilateral breast tissue was investigated). Group B is comprised of 87 female patients (*n* = 87) diagnosed with primary invasive breast carcinoma whose tumors have not been exposed to neoadjuvant chemotherapy (NAC) and collected postsurgically, while group C represents an independent sample of female patients (*n* = 43) diagnosed with invasive breast carcinoma whose samples were exposed to NAC preoperatively. Table 1 shows the clinicopathological features of sample groups B and C. The median patient age was 55 years (range: 31–82 years) and 50 years (range: 28–69 years) for groups B and C, respectively. Among the 87 patients in group B, 68 (78%) had invasive ductal carcinoma (IDC) and 14 (16%) had invasive lobular carcinoma (ILC), while in group C, the number of patients who had IDC is 34 (79%) and ILC is 4 (9%).

Tumor stage ranged from stage IIA to stage IIIC in group B and from stage IA to stage IIIB in group C (Table 1). Staging was established based on the American Joint Committee on Cancer 8th edition guidelines [30]. A total of seven (8%), 42 (49%) and 38 (44%) of the tumors were grade I, II and III, respectively, in group B, while one (2%), 23 (53%) and 19 (45%) of the tumors were grade I, II and III, respectively, in group C. In group B, 67 (77%) tumors that were estrogen receptor (ER) positive, 66 (76%) tumors were progesterone receptor (PR) positive, and seven (8%) cases had triple-negative disease (TNBC). In terms of molecular subtyping, 57 (66%) of the tumors were luminal A, whilst 22 (25%) were luminal B and one case (1%) was positive only for human epidermal growth factor receptor 2 (HER2+\ER−\PR−). In group C, 38 (88%) cases had tumors that were ER positive, 35 (81%) tumors were PR positive, and one case (2%) had TNBC. Furthermore, there were 28 (65%) luminal A tumor samples, 12 (28%) luminal B and two cases (5%) were HER2+\ER−\PR− only. Lymphovascular invasion was recorded in 52 (60%) of the cases in group B and in 25 (58%) of the cases in group C. Finally, the number of samples that were positive for axillary lymph nodes was 59 (70%) and 30 (70%) in groups B and C, respectively (Table 1).

### 2.2. Lamin B1 Immunoexpression Levels in Normal Breast Epithelium, Primary Invasive Breast Carcinoma Exposed or Not Exposed to NAC

**Figure 1:**
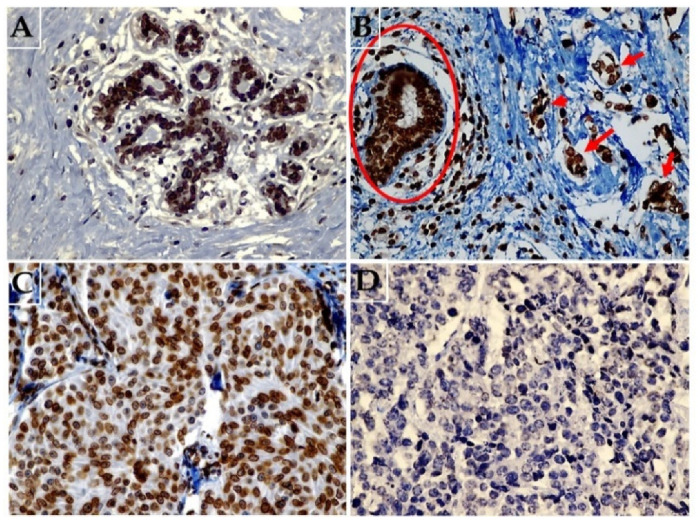
Representative images of immunohistochemical expression of lamin B1 in breast tissue. (**A**) Normal breast tissue showing diffuse strong expression of lamin B1 (original magnification: 400×). (**B**) Malignant breast cancer cells (arrows) not exposed to NAC and adjacent non-malignant breast duct (circle) both showing high lamin B1 expression (original magnification: 400×). (**C**) Malignant breast tissue exposed to NAC a with weak and focal expression of lamin B1 (original magnification: 400×), and (**D**) breast carcinoma tissue following exposure to NAC with no identifiable lamin B1 expression (original magnification: 400×).

**Figure 2:**
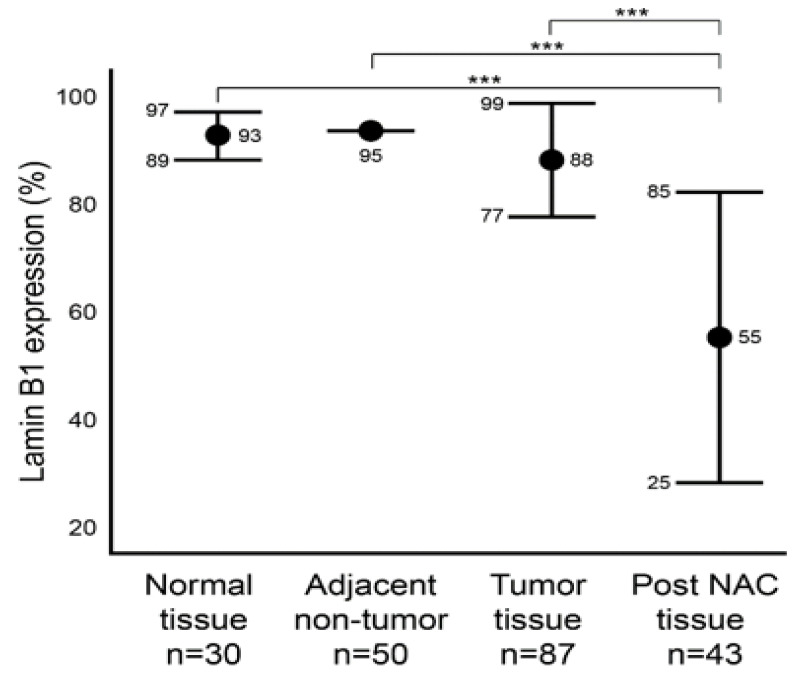
Comparison of lamin B1 protein expression levels within normal, adjacent non-tumor, tumor (not exposed to NAC), post NAC breast formalin-fixed paraffin-embedded (FFPE). Error bars show mean and ± 1 SD. Statistical differences were calculated using Mann-Whitney test. ***: *p* values < 0.001.

We first aimed to characterize the baseline level of lamin B1 protein expression in normal (Group A) and malignant (Group B) breast tissue samples utilizing IHC (Figure 1). In addition, we characterized the protein expression level of lamin B1 in the adjacent non-malignant tissue of group B. Group B is comprised of 87 tumor samples that were not exposed to NAC. Of these, 50 adjacent non-malignant tissues were examined. Among the normal breast epithelium samples (Group A), lamin B1 mean positive protein expression was 93% (range: 80–95%), while all the 50 adjacent non-tumor samples had a score of 95% lamin B1 positive staining (Figure 2). A mean of 88% (range: 50–95%) DAB-positive tumor cells was observed in the 87 tumor samples (Group B) (Figure 2). Remarkably, a significant decrease (*p* < 0.001) of lamin B1 protein expression was seen among the malignant cells within the post NAC samples at a mean of 55% (range 2–95%) (Figure 1 and Figure 2). Such observation suggests that lamin B1 expression in breast cancer is highly affected by exposure to NAC and could reflect the induction of TIS.

### 2.3. Association between Lamin B1 and Clinicopathological Characteristics of Patients with Invasive Breast Carcinoma

**Figure 3:**
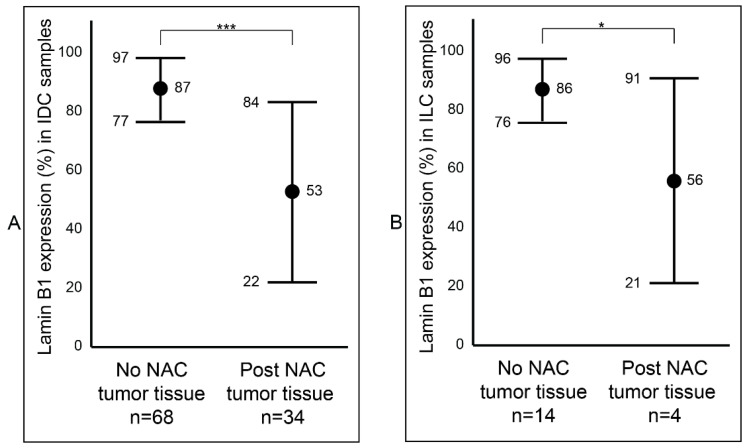
Assessment of lamin B1 protein expression between not exposed to NAC and post- NAC invasive breast carcinoma according to histopathological type. (**A**) In IDC samples. (**B**) In ILC samples. Error bars show mean and ± 1 SD. Statistical differences were calculated using Mann-Whitney test. ***: *p* value < 0.001; *: *p* value < 0.05.

**Figure 4:**
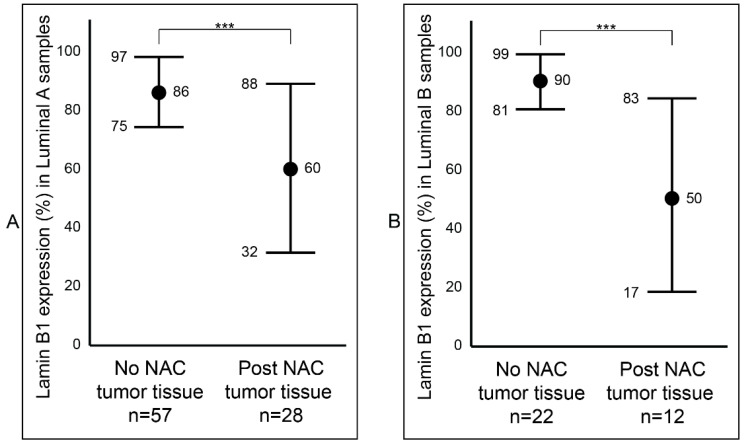
Comparison of lamin B1 protein expression between not exposed to NAC and post- NAC invasive breast carcinoma according to the molecular subtyping. (**A**) In luminal A samples. (**B**) In luminal B samples. Error bars show mean and ± 1 SD. Statistical differences were calculated using the Mann-Whitney test. ***: *p* value < 0.001.

After establishing a notable difference of lamin B1 protein expression between the samples that were not exposed to NAC (Group B) and the samples that were exposed to NAC (Group C) breast tumor samples, we wanted to determine if this difference was affected by tumor type (IDC and ILC) or luminal status (A and B). Among the IDC samples, we observed a statistically significant reduction (*p* value < 0.001: ***) of lamin B1 protein expression between the not exposed (mean 87%, range: 50–95%) and exposed to NAC (mean 53%, range: 2–95%) patients’ samples (Figure 3A and Figure S1). Similar findings were also seen among the ILC tumor samples: not exposed to NAC with a mean of 86% (range: 70–95%) and exposed to NAC with a mean of 56% (range: 5–85%) (Figure 3B and Figure S1). Furthermore, breast tumor samples that were identified as luminal A were examined and found to have a notable decrease of lamin B1 protein expression between not exposed to NAC (mean 86%, range: 50–95%) and post-NAC (mean 60%, range: 10–95%) (*p* value < 0.001: ***) (Figure 4A). As expected, we found a similar difference, albeit bigger, among luminal B malignant breast FFPE samples (Figure 4B).

### 2.4. Analysis of Lamin B1 Protein Expression in Association with the Clinicopathological Characteristics of Patients with Invasive Breast Carcinoma

As mentioned before, the status of lamin B1 protein expression in breast cancer has not been investigated. However, Wazir et al. investigated *LMNB1* gene expression in breast cancer (regardless of treatment status) and indicated no significant association between *LMNB1* mRNA in both tumor and adjacent non-tumor cells with several clinicopathological characteristics [23]. Subsequently, we sought to assess any potential association of lamin B1 protein expression in relation to breast cancer molecular subtyping, TNM stage, lymphovascular invasion and lymph node involvement. Among the cases that were not exposed to NAC (Group B) with luminal A status, the number of samples of low and high lamin B1 expression were almost similar, at 29 and 28, respectively (Table 2). Although there was almost double the number of cases with high expression of lamin B1 (*n* = 15) compared to low lamin B1 expression (*n* = 7) in tumors with luminal B status, these differences were not statistically significant (*p* value = 0.366) (Table 2). Similarly, we did not find any statistically significant difference between lamin B1 expression and TNM stage (*p* value = 0.506). Moreover, we observed a slight increase in the number of tumor samples that were not exposed to NAC that showed higher lamin B1 protein expression when there was positive lymphovascular invasion, albeit not statistically significant (*p* value = 0.565). Examining lamin B1 protein expression in terms of lymph node involvement within the not exposed to NAC samples did not show any statistical difference (*p* value = 0.112) (Table 2).

We also performed similar association analysis between lamin B1 protein expression and the clinicopathological data of the post-NAC samples (Group C). As expected, almost all the cases with luminal A or B status exhibited low lamin B1 IHC expression, but this was not statistically significant (*p* value = 0.771) (Table 3). Similar observations we noted when we tested the TNM stage were lymphovascular invasion and lymph node involvement of the post-NAC tumors; more than 90 of the cases showed lower lamin B1 expression, nevertheless, these findings were not significant (Stage: *p* value = 0.319; lymphovascular invasion: *p* value = 0.881; lymph node involvement: *p* value = 0.340). (Table 3).

### 2.5. Survival Analysis of Lamin B1 Expression in Invasive Breast Carcinoma

**Figure 5:**
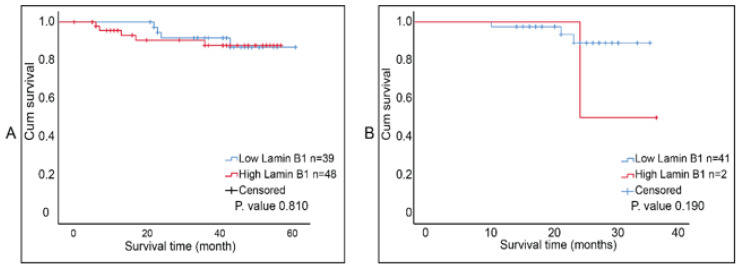
Kaplan Meier survival curves for lamin B1 protein expression in breast cancer samples. (**A**) Postsurgical untreated tumor samples (Group B, *n* = 87). (**B**) Post-NAC tumor samples (Group C, *n* = 43). Cut-off value was determined using ROC curve at 92%.

The report by Wazir et al. indicated that *LMNB1* mRNA expression levels in breast cancer decreased with declining clinical outcomes [23]. We investigated the correlation between lamin B1 protein expression and the overall survival of breast tumor samples that were not exposed to NAC and ones that were post-NAC. Although Kaplan–Meier survival curves (accompanied with log-rank test) showed that higher lamin B1 expression is associated with shorter survival time in both patients’ cohorts, this was not of statistical significance (Figure 5A,B). Therefore, we concluded that there was no prognostic value of lamin B1 expression based on our analysis and the studied breast cancer samples.

## 3. Discussion

TIS represents a fundamental response to a plethora of anticancer therapies [2,3,31]. While senescent tumor cells can be routinely identified in culture (and to a lesser extent in tumor-bearing animals) based on changes in the expression of several senescence-associated biomarkers, their detection in patient tumor samples continues to represent a significant challenge. This is due to several reasons, including: (*i*) the utilization of the canonical senescence biomarker SA-β-gal is limited to frozen tumor samples (rather than the more readily available fixed tumors), and the use of archived frozen samples (rather than flash frozen, fresh samples) is subject to error, since the activity of SA-β-gal might be altered [32]; (*ii*) the reliance on using a single senescence biomarker, an approach that is not recommended even for in vitro studies [33]; (*iii*) the studied sample size is small, limiting the ability to establish statistical correlation with the contribution of senescence induction to disease outcome [34,35]; and (*iv*) estimating the extent of senescence induction to only a single regimen of NAC rather than providing a comparison of the ability of different therapy approaches to induce senescence clinically [36]. Consequently, the characterization of senescence-associated biomarkers, including lamin B1, should facilitate the assessment of clinical senescence in breast cancer patients, allowing for the selection of more effective therapeutic approaches [32,34,37].

Degradation of lamin B1 has been established as a component of the senescent phenotype [21]. Loss of lamin B1 in senescent cells is part of wide-spectrum alterations in the nuclear landscape and is associated with significant changes in gene expression [22]. While the functional contribution of lamin B1 extends to maintaining nuclear stability, regulating DNA replication and gene transcription [15,16,18], its role as a senescence biomarker is of importance [17]. This is due to the increasing evidence that indicates that (*i*) TIS is an unfavorable response to therapy [38,39,40,41,42,43]; and (*ii*) the removal of senescent tumor cells that are produced is a consequence of exposure to common cancer therapeutics as a novel approach to mitigate cancer recurrence [44,45,46,47]. However, these efforts are largely hindered by the lack of reliable detection of TIS in clinical cancer samples, especially of the breast, where senescence-inducing therapy is often employed as both neoadjuvant and adjuvant treatment [48]. In this work, we aimed to characterize lamin B1 expression in breast cancer tissue and the change in protein expression levels that might occur in response to exposure to senescence-inducing chemotherapy.

Numerous studies have demonstrated the expression of lamin B1 in other malignant tumor tissue. For example, the relative expression of lamin B increases significantly in prostate cancer tissue samples in comparison to their normal prostate tissue counterparts (mean: 12.8% to 4.8%, respectively) [24]. Furthermore, proteomic analysis indicated that lamin B1 is significantly upregulated in hepatocellular carcinoma tissue and is circulating in the plasma of corresponding patients, which can be useful for the detection of early stages of liver cancer [26]. In a cohort of 71 gastric carcinoma samples, lamin B1 expression was relatively lower when compared to its expression in the stromal cells [49]. On the contrary, the immunohistochemical analysis of lamin B1 in 86 lung adenocarcinoma samples showed a higher expression level in comparison to 14 tumor-adjacent tissue samples [50]. Importantly, a large-scale tissue microarray analysis of 622 clear cell renal carcinoma (and their corresponding normal kidney tissue) revealed that lamin B1 is expressed in 80.87% of the tumor samples at a cut-off rate of ≥60% positive expression [27]. This evidence indicates that lamin B1 expression varies based on malignant tissue type. Our study has shown that lamin B1 protein expression levels were relatively high in both normal and malignant breast tissue.

In breast cancer, the report by Cotarelo et al. investigated the expression of SA-β-gal, the classical senescence marker, in frozen sections of invasive breast carcinoma tissue samples [37]. Interestingly, this work demonstrated that the majority of examined breast cancer samples (*n* = 129) showed some degree of staining with SA-β-gal, with luminal A and HER2+ subtypes exhibiting the highest rates of high-SA-β-gal staining [37]. Furthermore, Cotarelo et al. and colleagues have investigated lamin B1 protein expression using IHC in the breast samples, which was inversely correlated with SA-β-gal expression, indicative of its reliability as a senescence-associated marker [37]. However, their assessment of lamin B1 expression was limited to samples showing the highest SA-β-gal (instead of entire tissue sample), and there was no clear explanation of how lamin B1 expression was quantified, and the study did not include exposure to senescence-inducing chemotherapy as a study condition [37]. In comparison, our study investigated three different cohorts including patients with normal breast epithelium, patients with malignant breast tissue that was not exposed to senescence-inducing NAC, and patients with malignant breast tissue following exposure to senescence-inducing NAC. Our analysis showed that lamin B1 protein expression significantly decreases following exposure to NAC, consistent with previous reports that suggested that senescence is a potential response to chemotherapy in clinical cancer [34,35].We were not able to identify any previous evidence on the change in expression of lamin B1 following the exposure of breast cancer tissue to NAC. In vitro experiments confirmed that lamin B1 protein expression is downregulated in breast tumor cells upon exposure to NAC and senescence induction. For example, exposure of MDA-MB-231 TNBC cells to clinically relevant concentrations of doxorubicin resulted in a significant reduction in lamin B1 protein expression [44]. Moreover, lamin B1 appears to be more reliable in reflecting a senescent state in breast cancer following exposure to NAC than other established senescence-associated markers such as p21^Cip1^ and H3K9Me3 [51,52].

Our study has several limitations. First, despite that this is the first report to characterize lamin B1 protein expression in human normal and malignant breast tissue, the analysis could have benefited from increasing the sample size. Second, our sample selection criteria excluded patients with stage IV breast cancer, which could explain the relatively high survival rate observed in our sample. The rationale behind this selection was to include patients who will undergo surgery and whose postoperative samples will be available for pathological examination. Furthermore, postsurgical analysis was only performed on samples with incomplete pathological response to treatment, since patients whose tumors responded completely to NAC do not have sufficient tumor tissue for pathological analysis. Lastly, adjuvant therapy was not included in the survival analysis. All of these factors could explain the inability to observe a prognostic value of lamin B1. However, as mentioned previously, Wazir et al. indicated that the decrease of *LMNB1* mRNA expression levels was associated with worse clinical outcomes, suggesting that senescence induction might not be an advantageous consequence of cancer treatment. Finally, the reduction of lamin B1 protein expression levels in malignant breast tissue after the exposure to NAC can also be explained by a reduction in its gene expression, which has been reported as part of TIS [43], and thus our data reflect the downregulation of lamin B1 expression and not its degradation specifically.

Overall, our study indicates that lamin B1 protein expression levels are relatively high in normal and malignant breast tissue but that they undergo a dramatic reduction following exposure to cytotoxic chemotherapy indicative of TIS induction. However, it is noteworthy that should lamin B1 downregulation be used to identify senescence in clinical cancer samples, it must be combined with other senescence-associated biomarkers. This invites a more comprehensive identification of transcriptomic and proteomic signatures of TIS in different cancer types exposed to senescence-inducing therapy in order to generate a better understanding of the contribution of TIS to overall treatment outcomes.

## 4. Materials and Methods

### 4.1. Sample

This study sample consisted of three groups: the first group (Group A) represents 15 female patients (*n* = 15) who were admitted at Prince Hamza Hospital (PHH) and underwent bilateral breast reduction mammoplasty (*n* = 30). Breast tissue was collected bilaterally and confirmed to be pathologically-free. The second group consisted of 87 female patients (*n* = 87) that were diagnosed with different stages of primary non-metastatic invasive breast carcinoma undergoing modified radical mastectomy at the Jordan Royal Medical Services (JRMS) and PHH between the years 2017 and 2021. The inclusion criteria included (*i*) age between 18 and 90 years, and (*ii*) diagnosis of a breast cancer subtype (stages I-III); while the exclusion criteria included (*i*) patients with metastatic disease (stage IV), (*ii*) patients who received one form of neoadjuvant therapy (cytotoxic chemotherapy, hormonal, or targeted therapy) prior to surgery (subsequently, samples from this group are indicative of tumor baseline protein expression), (*iii*) patients diagnosed with ductal carcinoma in site (DCIS), and (*iv*) patients whose FFPE tumor tissue samples were unavailable. The third group (Group C) consisted of 43 female patients (*n* = 43) who received neoadjuvant therapy prior to surgical resection, and whose FFPE tumor tissue samples were collected between the years 2017 and 2021 from the Department of Pathology at JRMS. The criteria for inclusion included: (*i*) age between 18–90 years; (*ii*) diagnosis of a breast cancer subtype (stages I–III), (*iii*) receiving one type of NAC prior to undergoing surgical resection including: docetaxel, Adriamycin and cyclophosphamide (TAC), paclitaxel and doxorubicin plus cyclophosphamide (ACP), Adriamycin plus cyclophosphamide (AC), docetaxel plus cyclophosphamide (TC), 5 fluorouracil, epirubicin and cyclophosphamide (FEC) or 5-fluorouracil, epirubicin, cyclophosphamide followed by docetaxel (FEC+D) regardless of receiving other modes of therapy such as hormonal treatment or radiotherapy; (*iv*) developing partial pathological response to NAC. On the other hand, the exclusion criteria included (*i*) patients with metastatic disease (Stage IV), (*ii*) patients whose tumors developed complete pathological response (pCR) to NAC (and thus, have insufficient postsurgical tumor tissue for immunohistochemical evaluation), (*iii*) patients diagnosed with DCIS, and (*iv*) patients whose postsurgical FFPE tumor tissue samples were unavailable. The layout for the three groups is outlined in Supplementary Table S1 while the NAC treatment regimens received by patients of Group C are outlined in Supplementary Table S2.

### 4.2. Immunohistochemical Procedures

Immunostaining for lamin B1 was performed using the horseradish peroxidase-3,3′-diaminobenzidine (HRP-DAB) detection method as described in our previous publications [51]. Briefly, paraffin blocks of breast tissue were cut into 4 μm-thick slices using a microtome (Leica, RM2125RT, Germany) and put on a positively charged glass slide. Tissue sections were deparaffinized with fresh xylene solution for 10 min and rehydrated in a graded series of ethanol (100%, 95%, and 70%). Antigen retrieval was performed using a sodium citrate buffer (pH 6.0) for 60 min at 95–98 °C in a water bath and then cooled for 30 min at room temperature.

Tissue sections were incubated for 10 min with 3% hydrogen peroxide in deionized water following washing with phosphate-buffered saline (PBS). Then, the sections were permeabilized with 0.1% Triton X-100 in PBS for 15 min at room temperature. Next, sections were incubated with a blocking solution (PBS, 5% bovine serum albumin, 0.1% Tween 20 detergent) for 45 min to minimize non-specific binding and then incubated with the primary monoclonal mouse anti-human lamin B1 antibody (1:600, Novus biologicals, Cat. No. NBP2-59783, clone 4001, USA) for 2 h at room temperature. After washing in PBS, tissue sections were incubated with a ready-to-use rabbit anti-mouse linker (catalog number: QD430-XAKE; BioGenex, USA) for 45 min at room temperature. Subsequently, tissue samples were washed again and then incubated with an HRP-labeled polymer reagent (catalog number: QD430-XAKE; BioGenex, USA) for 35 min at room temperature. 3,3′-diaminobenzidine was used as a chromogen, and hematoxylin was used for counterstaining. Lastly, the stained sections were mounted in dibutyl phthalate in xylene (DPX) mounting media (#BCBX0183, Sigma, Germany).

Positive and negative tissue controls were performed routinely in each run to avoid false positive or negative results (Supplementary Figure S2). According to the manufacturer’s instructions, normal human colon tissue served as the positive control while omitting the lamin B1 antibody, which served as a negative control (Supplementary Figure S2). Four independent pathologists (H.A., N.A.S., E.A., and T.N.A) evaluated the immunoexpression level of lamin B1 on each slide by applying the semi-quantitative scoring system (the percentage of positive cells). A receiver operating characteristic (ROC) curve analysis was performed to determine the cut-off value for lamin B1 protein expression in breast cancer (Supplementary Figure S3). Accordingly, it was observed that the cutoff score for lamin B1 was 92.5%. Furthermore, the sensitivity and specificity of the lamin B1 were 90.4% and 41%, respectively. It is important to note that this is the first work that reported a cut-off value for lamin B1 immunoexpression in breast cancer tissue. Immunostaining of lamin B1 was observed at ×40 and ×200 magnifications and photographed using light microscopy (Olympus CX 41, Olympus, Tokyo, Japan).

### 4.3. Statistical Analysis

Data analyses were performed using the Pearson chi-square (X^2^) or Fisher’s exact non-parametric test to correlate the lamin B1 expression with clinicopathologic variables. Shapiro-Wilk and Kolmogorov-Smirnov tests were used to check whether the data were normally distributed. Given that the data were found not to appear to be a normal distribution, a Mann-Whitney test was used to evaluate the differences between the tested groups throughout this study. Survival curves were estimated using the Kaplan–Meier method and differences were tested by the log-rank test. All reported *p* values were 2-tailed and *p* ≤ 0.05 was considered statistically significant. All data were analyzed using SPSS (version 25).

## Figures and Tables

**Table 1:** Description of the clinicopathological characteristics of invasive breast cancer samples. Group B represents patients with invasive breast carcinoma that were not exposed to neoadjuvant chemotherapy (NAC). Group C represents patients with invasive breast carcinoma that were exposed to NAC. Groups B and C include unmatched sets of tumor samples from independent groups of patients. The table demonstrates the clinicopathological characteristics of groups B and C including histopathological subtyping, molecular subtyping, stage, grade, lymphovascular invasion and lymph node status. Other breast cancer subtypes include invasive metaplastic carcinoma, invasive mixed ductal and micropapillary carcinoma, invasive multifocal mixed ductal and micropapillary carcinoma, metaplastic breast cancer, mixed invasive lobular and ductal carcinoma. Abbreviations: ER: Estrogen Receptor; PR: Progesterone Receptor; HER2: Human Epidermal Growth Factor Receptor 2; TNBC: Triple Negative Breast Cancer. Other breast cancer subtypes include: invasive metaplastic carcinoma, invasive mixed ductal and micropapillary carcinoma, invasive multifocal mixed ductal and micropapillary carcinoma, metaplastic breast cancer, mixed invasive lobular and ductal carcinoma.

		Group B (*n* = 87) *n* (%)	Group C (*n* = 43) *n* (%)
Breast Cancer Subtype	IDC	68 (78%)	34 (79%)
	ILC	14 (16%)	4 (9%)
	Other	5 (6%)	5 (12%)
ER	Positive	67 (77%)	38 (88%)
	Negative	20 (23%)	5 (12%)
PR	Positive	66 (76%)	35 (81%)
	Negative	21 (24%)	8 (19%)
HER2	Positive	23 (26%)	14 (33%)
	Negative	64 (74%)	29 (67%)
Luminal A	ER+/PR+ & HER2−	57 (66%)	28 (65%)
Luminal B	ER+/PR+ & HER2+	22 (25%)	12 (28%)
HER2+	ER−/PR− & HER2+	1 (1%)	2 (5%)
TNBC	ER−/PR− & HER2−	7 (8%)	1 (2%)
Stage	Stage I	36 (41%)	22 (51%)
	Stage II	24 (28%)	12 (28%)
	Stage III	27 (31%)	9 (21%)
Grade	Grade 1	7 (8%)	1 (2%)
	Grade 2	42 (48%)	23 (%53)
	Grade 3	38 (44%)	19 (45%)
Lymphovascular invasion	Present	52 (60%)	25 (58%)
	Not identified	35 (40%)	18 (42%)
Lymph node involvement	Positive	59 (70%)	30 (70%)
	Negative	28 (30%)	13 (30%)

**Table 2:** Association of lamin B1 expression and clinicopathological variables of invasive breast carcinoma samples (Group B, *n* = 87). The table depicts statistical analysis of the association between lamin B1 expression in malignant breast cancer tissue and patients’ clinicopathological characteristics. Group B breast cancer samples were not exposed to neoadjuvant chemotherapy. *p* values were calculated using Pearson’s chi-square or Fisher’s exact non-parametric tests.

		Lamin B1 Low	Lamin B1 High	*p* Value
Luminal A (*n* = 57)	ER+/PR+ & HER2−	29 (51%)	28 (49%)	0.366
Luminal B (*n* = 22)	ER+/PR+ & HER2+	7 (32%)	15 (68%)	
HER2+ (*n* = 1)	ER−/PR− & HER2+	0 (0%)	1 (100%)	
TNBC (*n* = 7)	ER−/PR− & HER2−	3 (43%)	4 (57%)	
Stage	Stage I (*n* = 36)	14 (39%)	22 (61%)	0.506
	Stage II (*n* = 24)	13 (54%)	11 (46%)	
	Stage III (*n* = 27)	12 (44%)	15 (56%)	
Lymphovascular Invasion	Positive (*n* = 52)	22 (42%)	30 (58%)	0.565
	Negative (*n* = 35)	17 (49%)	18 (51%)	
Lymph node involvement	Positive (*n* = 28)	16 (57%)	12 (43%)	0.112
	Negative (*n* = 59)	23 (39%)	36 (61%)	

**Table 3:** Association of lamin B1 expression and clinicopathological variables of invasive breast carcinoma tissue after exposure to NAC (Group C, *n* = 43). The table depicts the statistical analysis of the association between lamin B1 expression in malignant breast cancer tissue and patients’ clinicopathological characteristics. Group C breast cancer samples were exposed to NAC. *p* values were calculated using Pearson’s Chi-square or Fisher’s exact non-parametric tests.

		Lamin B1 Low	Lamin B1 High	*p* Value
Luminal A (*n* = 28)	ER+/PR+ & HER2−	26 (93%)	2 (7%)	0.771
Luminal B (*n* = 12)	ER+/PR+ & HER2+	12 (100%)	0 (0%)	
HER2+ (*n* = 1)	ER−/PR− & HER2+	1 (100%)	0 (0%)	
TNBC (*n* = 2)	ER−/PR− & HER2−	2 (100%)	0 (0%)	
Stage	Stage I (*n* = 22)	22 (100%)	0 (0%)	0.319
	Stage II (*n* = 12)	11 (92%)	1 (8%)	
	Stage III (*n* = 9)	8 (89%)	1 (11%)	
Lymphovascular Invasion	Positive (*n* = 25)	24 (96%)	1 (4%)	0.881
	Negative (*n* = 18)	17 (94%)	1 (6%)	
Lymph node involvement	Positive (*n* = 30)	28 (93%)	2 (7%)	0.340
	Negative (*n* = 13)	13 (100%)	0 (0%)	

## Data Availability

The data included in this work is available on request from the corresponding author. Data is not publicly available due to patient privacy.

## Supplementary Materials

The following supporting information can be downloaded at: https://www.mdpi.com/article/10.3390/diagnostics12030609/s1, Table S1: Description of patients’ samples and their selection criteria; Table S2: Types of NAC received by each patient within Group C; Figure S1: IHC images of breast IDC and ILC without or with exposure to NAC; Figure S2: IHC images for positive and negative controls; Figure S3: Determination of lamin B1 immunohistochemical cut-off score.
