# Supplementary material for: The Expression of the Senescence-Associated Biomarker Lamin B1 in Human Breast Cancer
Source: Diagnostics (Basel). 2022 Feb 28;12(3):609. doi: 10.3390/diagnostics12030609 (PMC8947068; doi:10.3390/diagnostics12030609)
Supplement: Supplementary file 1 [file diagnostics-12-00609-s001.zip › diagnostics-1611518-supplementary.pdf]

	Description	n	Inclusion Criteria	Exclusion Criteria
**Group A**	Normal breast epithelium collected through mammoplasty	15 patients (30 breast tissue samples)		
**Group B**	Invasive breast carcinoma (collected through one form of mastectomy)	87 patients (87 malignant breast tissue samples with 50 samples with adjacent non-malignant cells)	(*i*) age: 18-90 years (*ii*) diagnosis of a breast cancer (stages I-III)	(*i*) stage IV patients (*ii*) patients who received one form of neoadjuvant therapy (*iii*) unavailability of FFPE samples
**Group C**	Invasive breast carcinoma exposed to one type of NAC (collected through one form of mastectomy)	43 patients	(*i*) age: 18-90 years (*ii*) diagnosis of breast cancer (stages I-III) (*iii*) receiving one type of NAC prior to undergoing surgical resection	(*i*) stage IV patients (*ii*) pCR to NAC (iii) unavailability of FFPE samples.

Table S1. Description of patients' samples and their selection criteria. The table describes the number of patients and corresponding tissue samples within the groups included in the study and the main inclusion and exclusion criteria that provided the basis for the sample selection process. NAC: neoadjuvant chemotherapy; pCR: complete pathological response, FFPR: formalin-fixed paraffin-embedded.

Patient	AC	AC+docetaxel	AC+ paclitaxel	FEC+ docetaxel	Trastuzumab	Pertuzumab
C1		×			-	
C2		×				
C3			×			
C4		×				
C5			×			
C6		×				
C7		×				
C8			×			
C9		×				
C10				×		
C11		×				
C12		×			×	×
C13		×				
C14		×				
C15	×					
C16		×				
C17		×			×	×
C18		×				
C19		×				
C20		×				
C21		×			×	×
C22			×			
C23				×		
C24		×			×	×
C25			×			
C26				×		
C27		×				×
C28		×				
C29				×		
C30				×		
C31		×				
C32		×				
C33		×				
C34		×				
C35				×	×	×
C36		×				
C37		×			×	×
C38		×				
C39				×		
C40		×				
C41		×				
C42		×				
C43		×			×	×

Table S2. **Types of NAC received by each patient within Group C**.

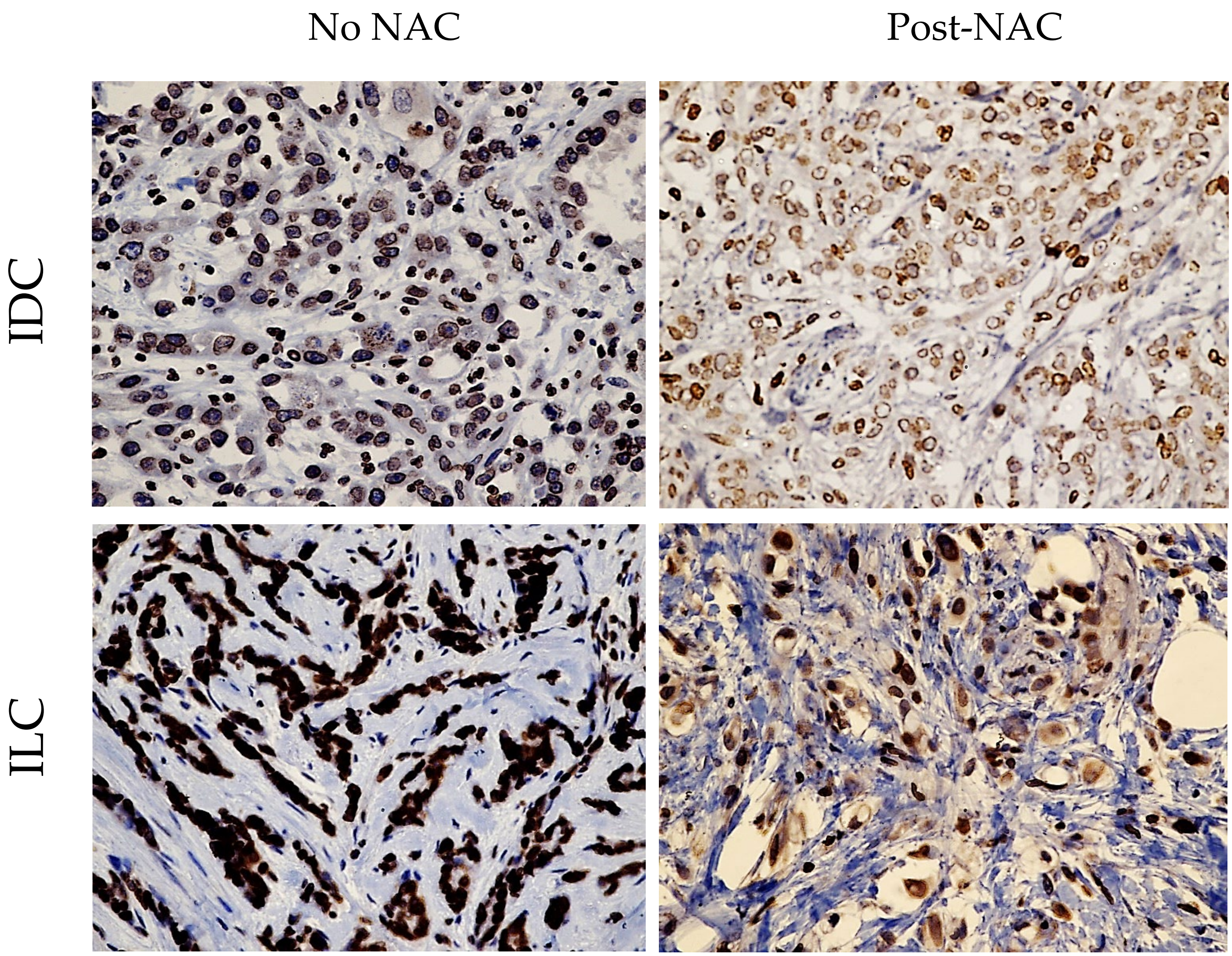

Figure S1. **IHC images of breast IDC and ILC without or with exposure to NAC.** Representative images of lamin B1 protein expression in invasive ductal carcinoma and invasive lobular carcinoma of the breast in samples from patients whose tumors were not exposed to NAC (Group B) and patients whose tumors were exposed to NAC prior to surgery (Group C). Note the high expression in group B compared to group C (Original magnification 400x).

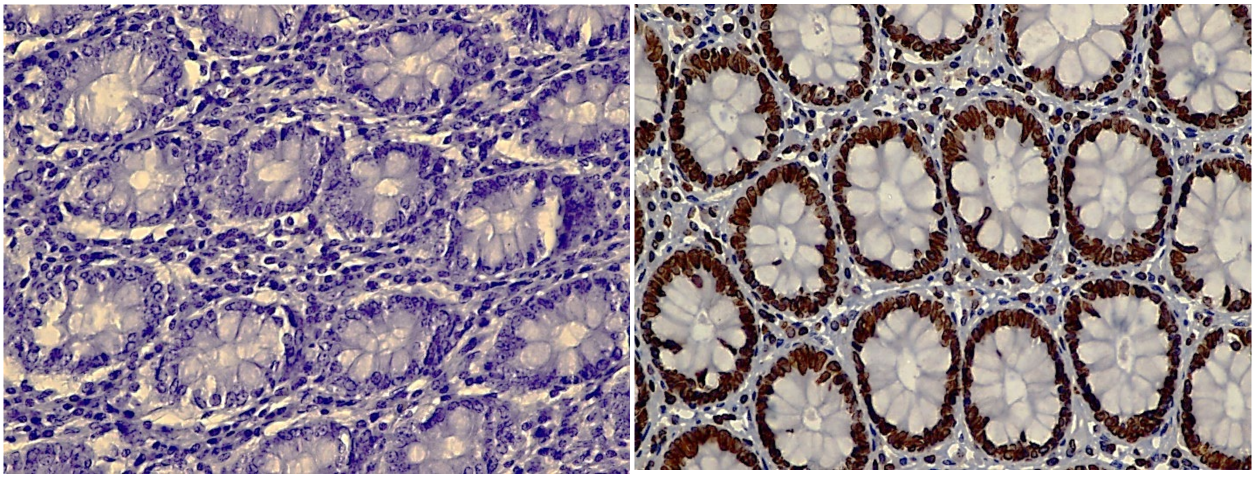

Figure S2. **IHC images for positive and negative controls.** Immunohistochemical staining of (A) negative and (B) positive controls (B) for lamin B1 nuclear expression in normal colonic mucosa (Original magnification 400x).

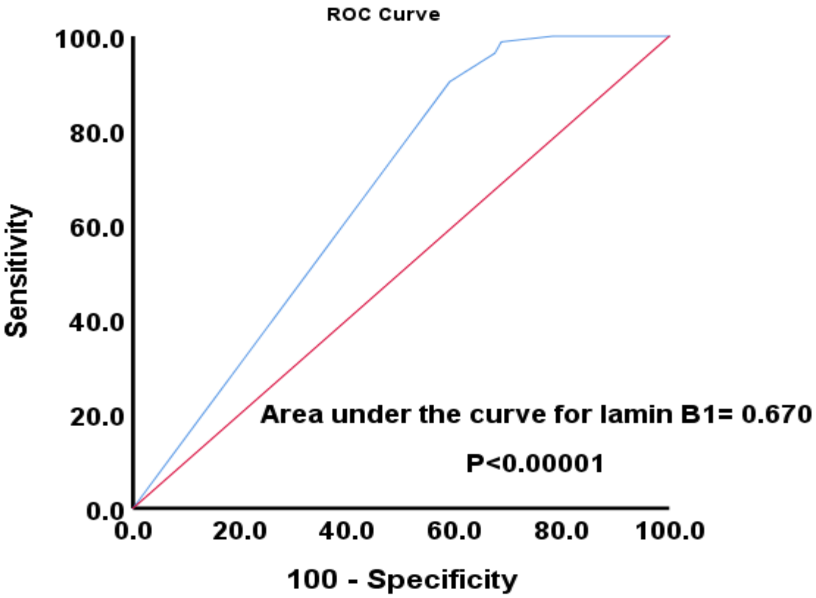

Area Under the Curve

Test Result Variable(s): Expression levels of malignant and benign groups

Area	Std. Error[a]	Asymptotic Sig.[b]	Asymptotic 95% Confidence Interval	
			Lower Bound	Upper Bound
.670	.042	<.00001	.587	.753

Figure S3. **Determination of lamin B1 immunohistochemical cut-off score**. Receiver operating characteristic (ROC) curve analysis was performed to determine the cut-off

point for lamin B1 expression in invasive breast cancer. The cut-off score for lamin B1 was 92.5%. The area under the ROC curve for lamin B1 lies with two-sided 95% confidence intervals was 0.670. An area of 0.5 is considered a non-discriminating test whereas an area of 1.0 is considered an ideal diagnostic test. Benign and malignant disease was considered a state variable. A ROC curve was created and calculated using SPSS 25 software.
